# Selection and Identification of a DNA Aptamer for Multidrug-Resistant *Acinetobacter baumannii* Using an In-House Cell-SELEX Methodology

**DOI:** 10.3389/fcimb.2022.818737

**Published:** 2022-06-30

**Authors:** Marina Farrel Côrtes, Taniela Marli Bes, Beatriz Ribeiro Deo, Beatriz Barbosa dos Anjos, Andrés Jimenez Galisteo, Ester Cerdeira Sabino, Carlos Santos, Silvia Figueiredo Costa

**Affiliations:** ^1^ Instituto de Medicina Tropical e Departamento de Moléstias Infecciosas e Parasitarias da Faculdade de Medicina da Universidade de São Paulo, São Paulo, Brazil; ^2^ Biologia Molecular, Clinimol Diag Molecular, Sao Paulo, Brazil

**Keywords:** aptamer, *Acinetobacter banumannii*, Cell-SELEX, protocol, multidrug resistance

## Abstract

Infections caused by multidrug-resistant *A. baumannii* are a worldwide health concern with high mortality rates. Rapid identification of this infectious agent is critical as it can easily spread with difficult or no options for treatment. In this context, the development of reliable and economically viable detection and therapeutic methodologies are still challenging. One of the promising solutions is the development of nucleic acid aptamers capable of interacting with bacteria. These aptamers can be used for specific recognition of infectious agents as well as for blocking their functions. Cell-SELEX technology currently allows the selection and identification of aptamers and is flexible enough to target molecules present in an entire bacterial cell without their prior knowledge. However, the aptamer technology is still facing many challenges, such as the complexity of the screening process. Here, we describe the selection and identification of a new aptamer A01, using an in-house whole-cell SELEX-based methodology, against multi-resistant *Acinetobacter baumannii*, with rapid execution and low cost. In addition, this protocol allowed the identification of the aptamer A01 with the whole *A. baumannii* cell as a target. The aptamer A01 demonstrated a binding preference to *A. baumannii* when compared to *K. pneumoniae*, *C. albicans*, and *S. aureus* in fluorescence assays. Although the time-kill assay did not show an effect on bacterial growth, the potential bactericidal or bacteriostatic cannot be totally discarded. The new categorized aptamer (A01) displayed a significant binding affinity to MDR *A. baumannii*.

## Introduction

The current concern of modern biotechnology is the search for new low-cost approaches to early diagnose and satisfactory treat infectious diseases (Afzal et al., 2016). Aptamers might be an answer to this problem. They are an innovative proposal that can be used for specific recognition and treatment of infectious diseases by several agents. They are single-stranded oligonucleotides of high affinity and specificity for organic molecules that can be obtained through the Systematic Evolution of Ligands by Exponential Enrichment (SELEX) technique. The search for receptors based on the use of aptamers aims to find functional nucleic acids that recognize a single target, minimizing cross-reactivity. High specificity is important when the purpose is to identify infectious diseases (Ulrich et al., 2005). Bacterial aptamers are mainly designed to target *Mycobacterium tuberculosis*, *Escherichia coli*, *Staphylococcus aureus*, *Listeria monocytogenes*, and *Salmonella* (Li et al., 2020).

First described in 1990, SELEX is an experimental procedure to identify aptamers. Characteristically, it starts with large libraries of 10^14–15^ random sequences of a combinatorial chemistry for virtual detection of any molecular target (Tuerk and Gold, 1990).

*Acinetobacter baumannii* is a challenging organism frequently found in hospital settings and it is associated with higher rates of morbidity and mortality mainly in critical care patients. *A. baumannii* frequently presents increased resistance to commonly used antibiotics, requiring broad spectrum antibiotics (Boucher et al., 2009). Currently, there are no treatment options to pan-resistant *A. baumannii* infections (Boucher et al., 2009). In this study we are using the whole *A. baumannii* cell as the aptamer target.

Here we describe the selection and identification of a new aptamer A01 using an in-house methodology, whole cell SELEX-based, against multi-resistant *A. baumannii*, as well as the prospects of applying nucleic acid aptamers for the development of novel detection system with rapid execution and low cost. The process we describe in this paper may be useful for a range of molecules and can be used to select aptamers against different types of cell targets.

## Materials and Methods

### Bacterial Samples

We first used four multi-drug resistant (MDR) *A. baumannii* carrying carbapenemases isolated between 2011 and 2013 of two different lineages according to MLST (Multilocus Sequence Typing). The resistance profiles of the isolates are shown in **Table 1**. The bacterial isolates of this study were previously sequenced and stored in the bacteriology laboratory (LIM49) biobank. These isolates have been phenotypically and genotypically characterized in previous studies (Leite et al., 2016; de Maio Carrillho et al., 2017). During the selection, aptamers were also tested on a clinical isolate of *Klebsiella pneumoniae*, as negative control. For the fluorescent assays *Candida albicans* and *Staphylococcus aureus* were also used as negative control.

**Table 1:** Resistance profile of *A. baumannii* isolates and the *K. penumoniae* K1375 used in this study.

	Whole genome sequencing							MIC	
Isolate	MLST	AMG	Beta-Lactam	Macrolide	Phenicol	SFM	TMP	Colistin	Meropenem
A552		–	*blaADC-25*	*mph (E)*	*-*	*-*	*-*	2	>8
	ST317		*blaOXA-23*	*msr (E)*					
			*blaOXA-88*	* *					
A891		*aadA1*	*blaADC-25*	*-*	*floR*	*sul2*	*dfrA1*	2	>8
	ST79	*aph(3’)-VIa*	*blaOXA-23*						
		*strA*	*blaOXA-65*						
		*strB*	*blaTEM-1^a^*						
A895		*aadA1*	*blaADC-25*		*floR*	*sul2*	*dfrA1*	1	>16
	ST79	*aph(3’)-VIa*	*blaOXA-23*	*mph (E)*					
		*strA*	*blaOXA-194*	*msr (E)*					
		*strB*	*blaTEM-1^a^*	* *					
A1013		*aadA1*		*-*	*-*	*sul2*	*dfrA1*	256	>8
	ST79	*strA*	*blaTEM-1^a^*						
		*strB*	* *						
K1375		*rmtB*	*blaTEM-1B*	* *	* *	*sul1*	*dfrA12*		
	ST258	*aac(3)-IId*	*blaCTX-M-14*	*-*	–	*sul2*	*OqxAB*	32	16

MLST, Multilocus Sequence Typing; ST, sequence Typing; AMG, Aminoglycoside; SFM, Sulfonamide; TMP, Trimethoprim; MIC, Minimal inhibitory concentration.

### Random DNA Library

The DNA aptamers chemically synthesized library consists of a sequence of 35 random nucleotides flanked by constant sequences that served as template for primers APT-F CAGGGGACGCACCAAGG and APT-R ATCACGCAGCACGCGGGTCATGG in the amplification reactions: 5’CAGGGGACGCACCAAGG-N35-CCATGACCCGCGTGCTGCGTGAT -3’ (75 nucleotides). The library and primers were obtained from IDT (Coralville, IA, USA) and resuspended to a final concentration of 10 µM in distilled water.

### Systematic Evolution of Ligands by Exponential Enrichment SELEX

Bacterial cultures were grown on LB agar plates (BD, Franklin Lakes, New Jersey, USA) and a pool of five *A. baumannii* isolates were standardized at 3.8 McFarland in saline solution (0.45%). The cells were then washed 3 times with saline by centrifugation at 6,000 rpm for 5 min. Subsequently, 100µl of the DNA aptamer library diluted in TE buffer (final concentration at 1µM) was denatured at 95°C for 5 min followed by immediate cooling on ice for 10 min prior to selection. In the first round of selection, the initial library was incubated with 10^7^ bacterial cells at room temperature for 25 min in 700µl of binding buffer (PBS 1x, Tween 20 0.05%, MgCl_2_ 1mM, and bovine serum albumin 0.2%) with gentle shaking (150 rpm). After the ligation reaction, unbound aptamers were removed after 3 washes in wash buffer (PBS 1x, Tween 20 0.05%, MgCl_2_ 1mM) by centrifugation at 6,000 rpm for 5 min. To elute the bacterial cell-bound aptamers, the cells were resuspended in 50 µl sterile TE, heated to 95°C for 10 min, and cooled on ice for 5 min. The cell suspension was then centrifuged at 6,000 rpm for 5 min and the supernatant stored at -20°C. The first round of SELEX was confirmed by PCR in a total reaction volume of 25 µl: 2.5 µl buffer, 2.5 µl MgCl_2_ 50mM, 0.75 µl dNTP mix 10 µM, 0.5 µl primers mix 10µM, 0.4µl Taq DNA Polymerase, recombinant (Invitrogen, Carlsbad, CA, USA), 2µl template DNA and water under the conditions: 95°C for 5 min followed by 20 cycles of 95°C for 10 sec, 60°C for 5 sec, 72°C for 4 sec, and a final extension of 72°C for 1 min. The size and purity of the PCR product was confirmed by 3% agarose gel electrophoresis dyed with SYBR SAFE (Invitrogen, Carlsbad,CA, USA). To generate single stranded DNA molecules, 2 µl of the product was used as template for asymmetric PCR with only one primer: 2.5 µl buffer, 2.5 µl MgCl_2_ 50mM, 0.75 µl dNTP mix 10 µM, 3.5 µl primer (forward or reverse) 10µM, 0.3µl recombinant taq (Invitrogen, Carlsbad,CA, USA), 2µl template DNA, and water to complete 25 µl. Four 25 µl asymmetric PCR reactions were performed totaling 100 µl for the next round of selection.

After approximately four rounds, the amount of leftover impurities resulting from incubation with the cells and PCR reagents made the following PCRs impractical. Therefore, the aptamer band (of 75 bp) was cut off from the agarose gel, resuspended in 1 ml of pure water and incubated at 90°C until complete dissolution. Then, a 10^-5^ dilution was performed to serve as a template for symmetric PCR and moved on to the next round. In addition, after the fourth round of aptamer and cell incubation, the number of washes with the washing buffer was increased from three to five, intending to eliminate as much unwanted PCR products as possible.

### Aptamer Identification

After at least 7 rounds, when single-stranded DNA aptamers bound to bacterial cells dominated the DNA pool, they were then cloned into the pGEM T-easy vector (Promega, Madison, WI, USA) as per manufacturer recommendations. The DNA of pGEM containing the insert was transferred into the chemically competent *E. coli* DH5α: 1ml of an overnight culture in LB broth was inoculated in 100mL of fresh LB until it reached an OD600nm 0.4-0.5 followed by ice incubation for 20 min. The culture was centrifuged by 4000 rpm for 15 min at 4°C, resuspended in 20ml of cold CaCl_2_, and incubated for 20 min on ice. Next, the culture was centrifuged, the pellet was washed four times with 5mL of cold CaCl_2_, and finally resuspended in 300µL of CaCl_2_ and incubated for 2 h on ice. 10µL of the ligase pGEM reaction was added and incubated for an additional 30 min, followed by 2 min at 52°C, and 1 mL of cold LB was added and incubated for 1 h at 37°C with gentle shaking (150 rpm). 200µL were plated in LB agar supplemented with ampicillin 100µg/mL (Gibco, Carlsbad, CA, USA). DNA of the colonies grown in the presence of the antibiotic was extracted by thermal lysis procedure (Pacheco et al., 1997). The amplification was performed with M13 primers (M13F 5’-CACGACGTTGTAAAACGAC-3 ‘ and M13R 5’-CAGGAAACAGCTATGACC-3’) targeting the multiple cloning site of the plasmid and the PCR product was purified using illustra GFX PCR DNA and Gel Band Purification Kit (GE Healthcare, Amersham Place, UK) and sequenced by Sanger MegaBACE 1000 (ABI 3730 DNA Analyzer; Applied Biosystems, Alameda, CA) using both M13F and M13R primers to confirm the aptamer sequence.

### Secondary Structure Prediction

The aptamer secondary structure was predicted using the RNAstructure tools version 6.0.1 (Bellaousov et al., 2013)

### Real-Time PCR Reaction (RT-PCR)

A RT-PCR reaction was performed to confirm the presence of aptamers by comparing the melting curve with the initial library curve, using the CFX96 device (BioRad). The PCR reaction was performed using 2 µl of Gotaq PCR (Promega) 0.2 µl EVAGREEN (Biotium), 0.4 µl primers, 2 µl template DNA, and 5.4 µl TE buffer under the following conditions: 94°C for 4min followed by 30 cycles of 95°C for 10 sec and 60°C for 5 sec.

Once identified, the aptamer A01 was chemically synthetized by IDT (Coralville, IA, USA) for *in vitro* testing.

### Fluorescence Assays

The binding specificity of A01 was analyzed with *A. baumannii* cells and *K. pneumoniae* or *S. aureus* as negative control in a fluorimeter. First, the aptamers were labeled with fluorescein 6-FAM by means of a PCR reaction as described above with primers labeled with 5’ 6-FAM (IDT, Coralville, USA). The labeled aptamers were incubated for 45 minutes, protected from light, with the bacterial cells. Afterwards, the cells were washed twice with PBS, 100µl were applied in 96-well plates and the material was read in a 530nm emission filter and 488nm excitation (FilterMax F5, Molecular Devices). The fluorescence after incubation of each isolate was normalized using the fluorescence value of each bacterial cell without the fluorescent aptamer. This experiment was performed in duplicate and repeated 6 times.

Flow cytometry was performed to assess the binding preference of the aptamers to *A. baumannii* cells and *K. pneumoniae* or *C. albicans*. Bacterial cells were incubated for 45 min with the labeled aptamers, as in the process described above, washed twice with FACSFLOW (BD) and resuspended in 1ml of FACSFLOW for analysis in the FORTESSA cytometer (BD). Data were collected using BD FACSDIVA^®^ (BD Biosciences^®^) software and analyzed in Flowjo V10^®^.

Additionally, after incubation of the bacterial with the fluorescent aptamers, the cells were observed on a fluorescent microscope Zeiss 466300 using a 100x objective and on the Zeiss LSM 780-NLO confocal microscope.

### Aptamer *In Vitro* Testing

25µl of TE or aptamer solution in different concentrations (10µM, 1µM, 0.1µM, and 0.01µM) was added to a bacterial layer of *A. baumannii* at 0.5 McFarland with and without thiourea at 5µM in MH agar. Additionally, a time-kill assay was conducted as described by (Petersen et al., 2006)). Briefly, a suspension of the bacteria at 0.5 McFarland was diluted at a 1:100 ratio in Mueller–Hinton broth (MHB) with aptamer at 0.5µM or TE (control) with and without thiourea at 0.05M for DNase inhibition and incubated at 37°C with 250 rpm shaking. Aliquots were removed at 0, 2, 4, 6, and 24 h post-inoculation and serially diluted in saline solution for UFC counting on trypticase soy agar (TSA) plates. Three biological experiments were performed for each condition.

### Statistical Analyses

Statistical analyses were based on the nonparametric T test using the Mann Whitney test and Wilcoxon test on GraphPad prism 8.3.

## Results

### SELEX Method

**Figure 1:**
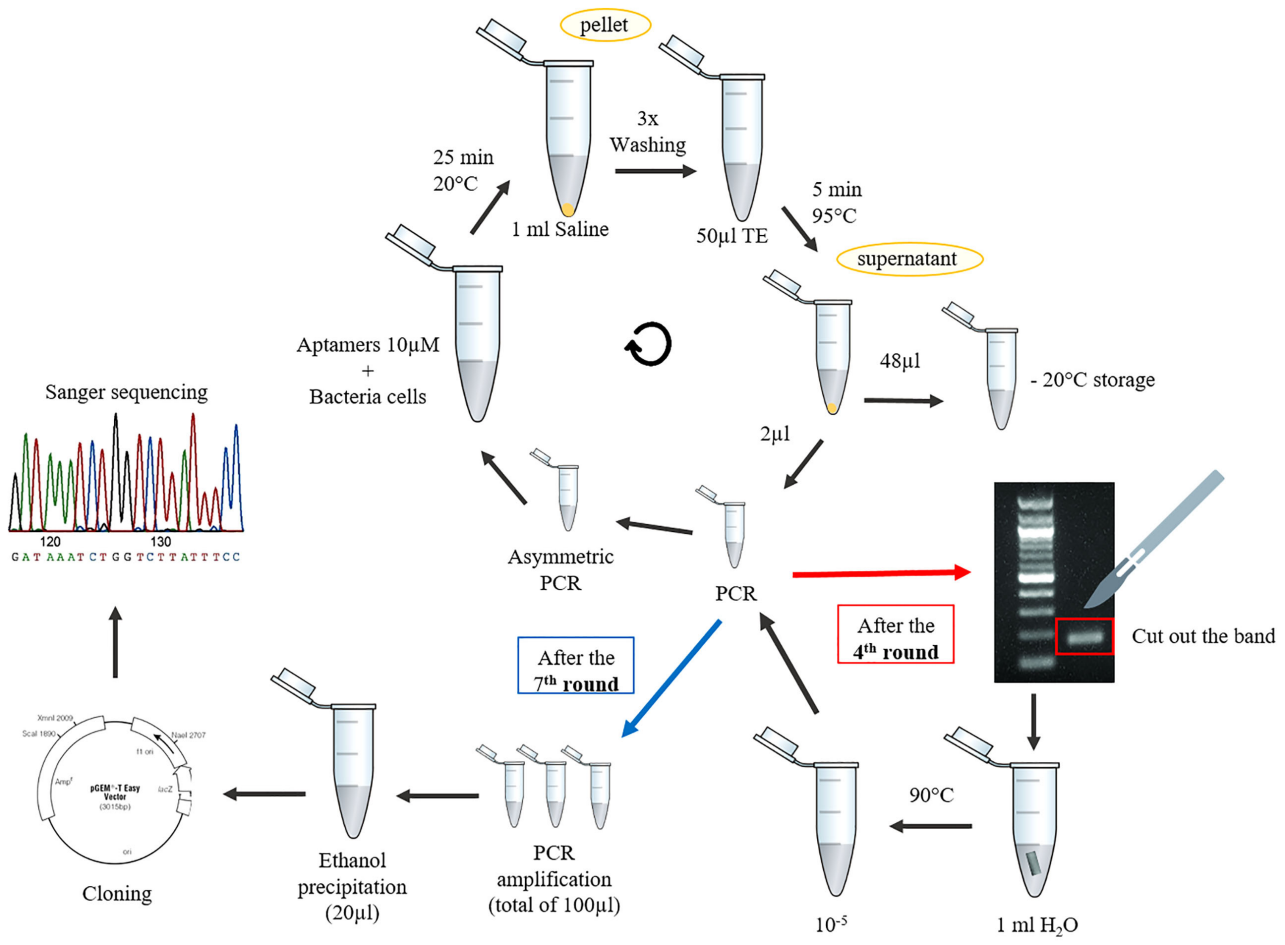
Steps of the Cell-SELEX protocol proposed in this paper.

**Figure 2:**
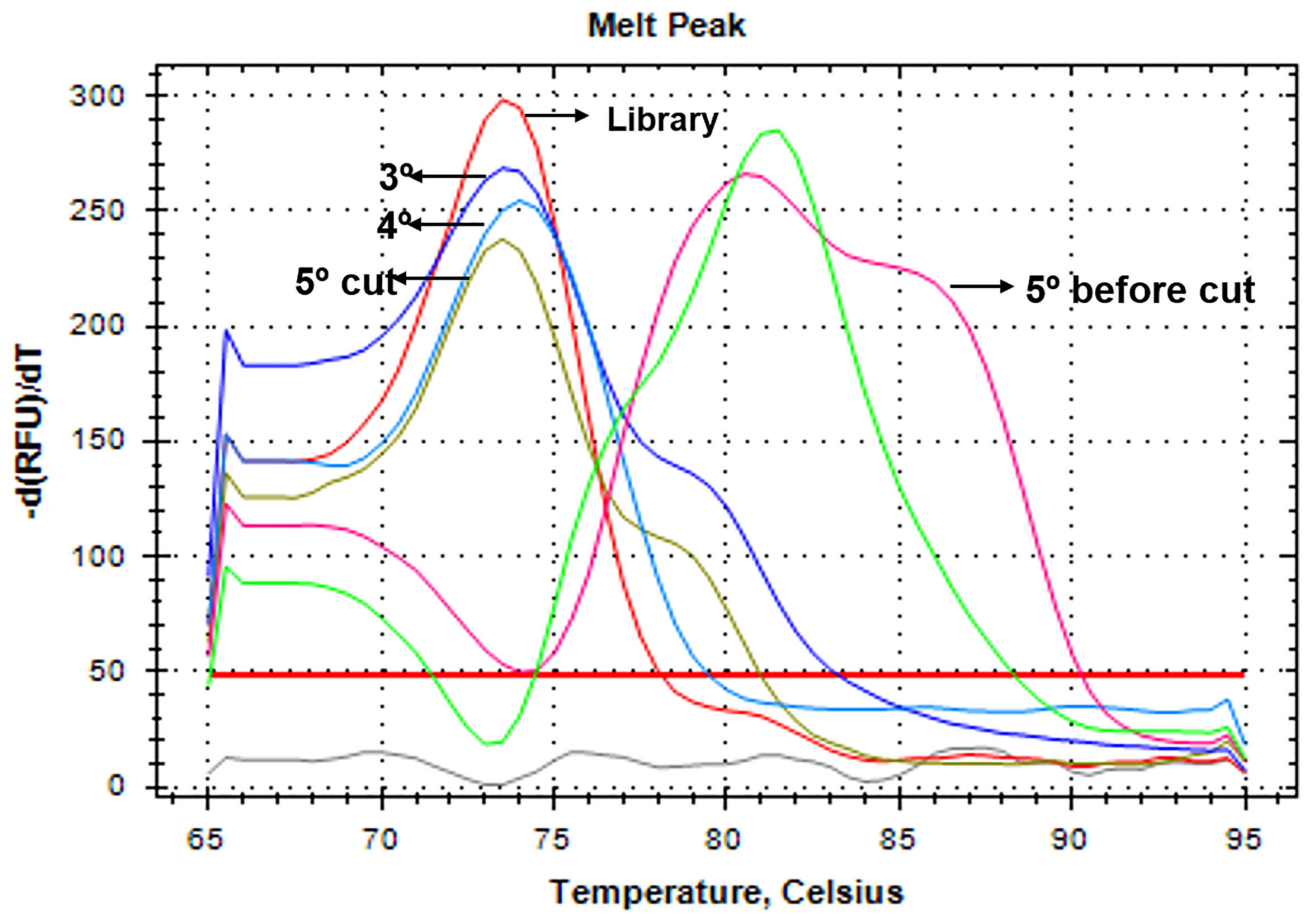
Melting curve of the Real time PCR performed after SELEX rounds. The positive control (library) is shown in red, and the SELEX round is shown with a black arrow. In pink, the 5^th^ round before the gel and in brown the same 5^th^ round but after cutting the band form the agarose gel, restoring the right peak.

Different SELEX protocols (Sefah et al., 2010; Kim et al., 2013; Heiat et al., 2017) were tested, combined, and optimized to arrive at a less expensive and equipment-intensive methodology. The standardized aptamer whole-cell-targeting SELEX technique is summarized in **Figure 1**. We recommend performing negative selections after the second, fourth, and last round of SELEX to ensure the selection of specific aptamers. It consists in the incubation with negative control cells and amplification of the aptamers in the supernatant that did not bind to the cells after the first wash. The presence of aptamers after the SELEX rounds were confirmed using a Real time PCR after each round. The melt curve ratified the need to cut the band from the agarose gel once the melting peak changes with the accumulation of unspecific amplification after the fourth round and then returns to the peak similar to the control when cut from the gel (**Figure 2**).

The average cost of this methodology is summarized in **Table 2**. Notably, the DH5α colony containing pGEM carrying the aptamer was confirmed by PCR using APT-F and APT-R primers. Since aptamers are small fragments and may not interfere in the β-galactosidase gene function, the blue/white colony selection of pGEM was not used.

**Table 2:** Average cost for aptamer selection and identification using the methodology proposed here totaling $301.5.

	Cost	1 reaction cost	Average cost for 1 aptamer
Random library	$75	$75	$75
Forward and Reverse primers (APT and M13)	$42	–	$42
taq polymerase	$65	–	$65
PCR purification kit (100 reactions)	$350	$3.5	~$7
pGEM T easy (20 reactions)	$270	$13.5	$13.5
Sanger sequencing (2 reactions)	$14	$7	$14
Aptamer synthetization	$75	–	$75
Ampicillin 200mg	$43	–	< $5
buffers and culture medium	$300	–	< $5

### Aptamer Identification

**Figure 3:**
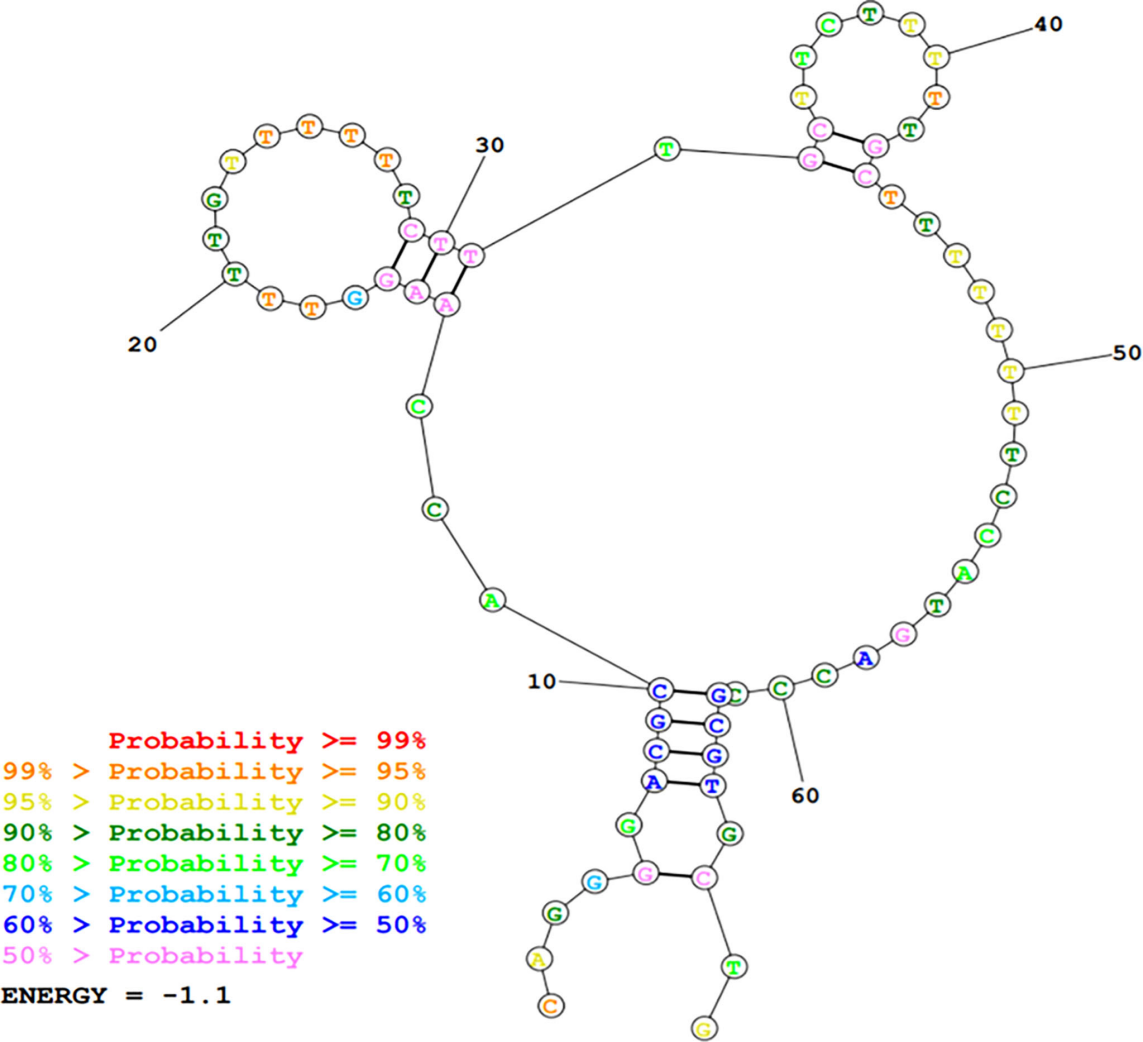
DNA secondary structure prediction of A01 using MaxExpect results structure of RNAstructure software package.

Sanger sequencing revealed the following sequence of A01 flanked by the APT-primers: 5’ CAGGGGACGCACCAAGG-TTTTGTTTTTTCTTTGCTTCTTTTTGCTTTTTTTT-CCATGACCCGCGTGCTGCGTGA 3’. The RNAstructure MaxExpect prediction generated a structure composed of highly probable A01 base pairs (**Figure 3**).

### Fluorescence Assays

**Figure 4:**
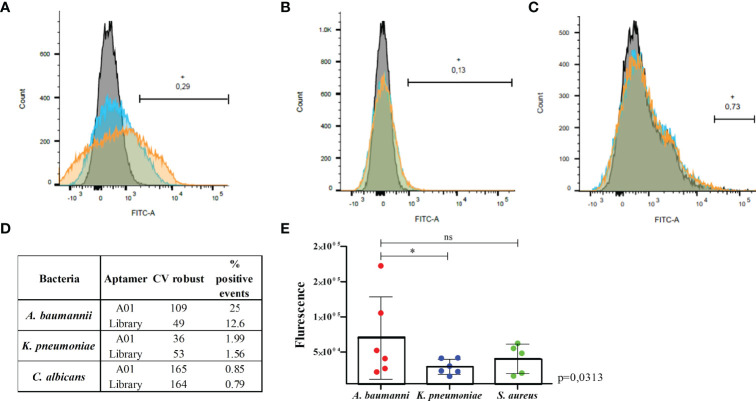
Fluorescence assays for A01 aptamer with *A. baumannii* compared to *K. pneumoniae*, *C. albicans* or *S. aureus* cells. Binding of the A01 aptamer (orange) relative to the starting library (blue) to *A. baumannii* **(A)**, *K. pneumoniae* **(B)** and *C. albicans* **(C)**. The fluorescence of cells without aptamer are indicated in grey. The black line indicates the number of positive events beyond the control (cells without aptamer) of the aptamer A01. CV robust and % of positive events are shown in the table **(D)**. Fluorimeter assay **(E)** for the aptamer A01 incubated with *A. baumannii* (red), *K. pneumoniae* (blue) or *S. aureus* (green). The average of each of the 6 experiments is shown in the graphics. * significant p value; ns, non sigificant p value. Wilcoxon test was applied with p value = 0.031 when *A. baumannii* was compared to *K. pneumoniae* and 0.537 when compared to *S. aureus*.

**Figure 5:**
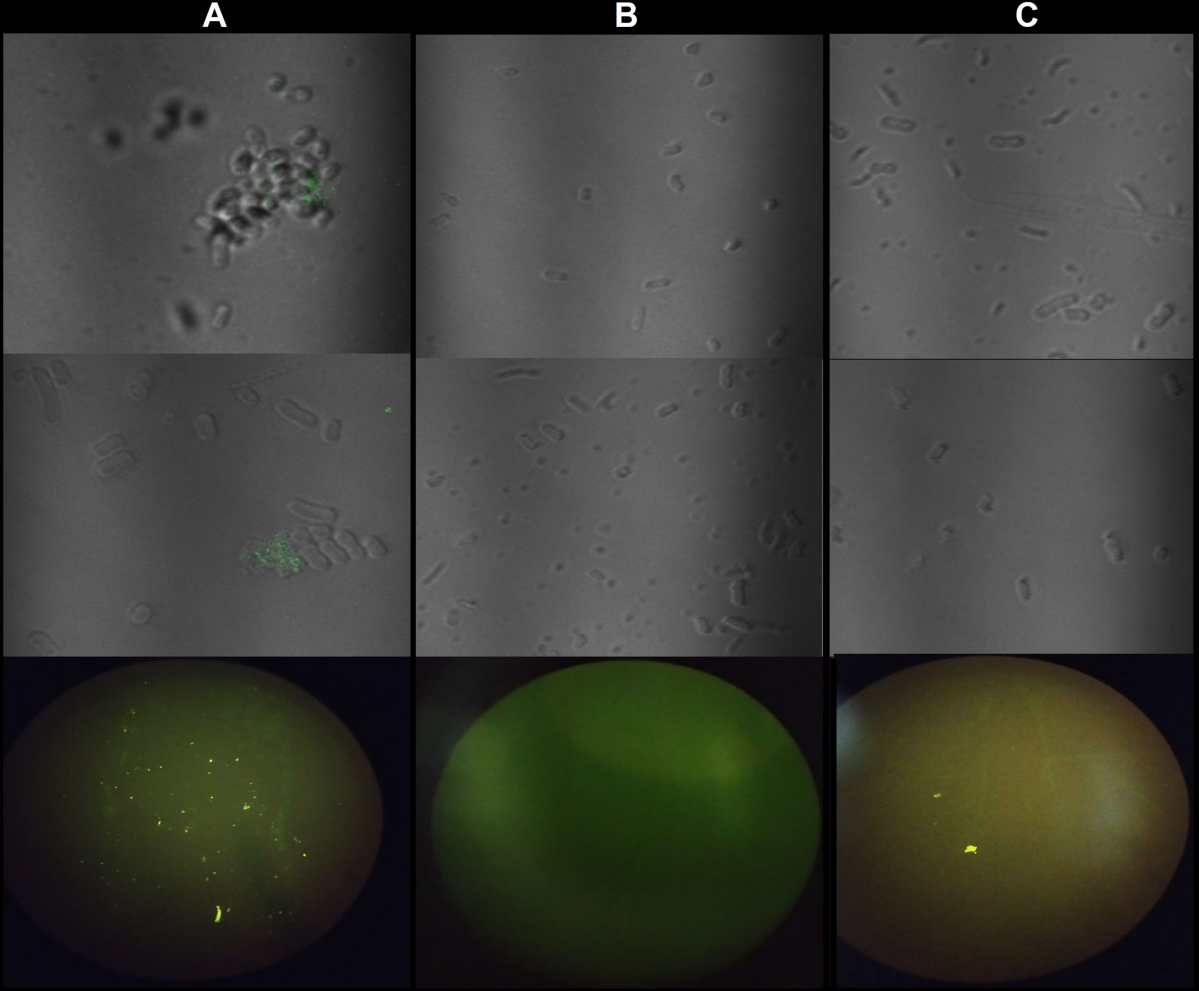
Fluorescence microscopy for A01 aptamer with *A. baumannii* and *K. pneumoniae* cells. **(A)** A01 incubated with to *A. baumannii* cells. **(B)** A01 incubated with *K. pneumoniae* cells. **(C)** cells without aptamer. On the top: Confocal microscopy from two different points on the slide. Bottom: Fluorescence microscopy.

The flow cytometer assay shows a greater shift in the fluorescence peak for *A. baumannii* cells when incubated with the A01 aptamer (**Figure 4A** orange) than with the library (**Figure 4A** blue) and bacterial cells alone (negative control; **Figure 4A** grey). The percentage of positive events above the negative control was twice as large for A01 compared to the library (25% and 12.66% respectively; **Figure 4D**). These differences are not evidenced for *K. pneumoniae* (**Figure 4B**) nor *C. albicans* (**Figure 4C**). The fluorimeter assay displays a significantly higher fluorescence when FAM-labeled A01 was incubated with *A. baumannii* cells, as to when incubated with *K. pneumoniae* or *S. aureus* cells (**Figure 4E**). Microcopy evidenced fluorescence spots when the aptamer is incubated with *A. baumannii* rather than *K. pneumoniae* (**Figure 5**).

### Aptamer *In Vitro* Testing

**Figure 6:**
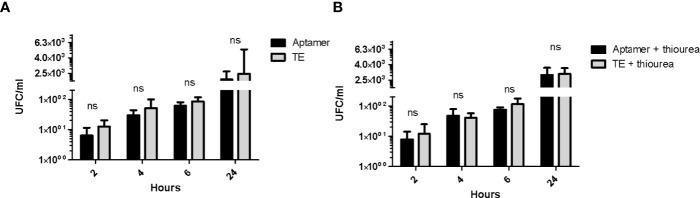
Time-kill kinetics assay of *A. baumannii* with the A01 aptamer at 0.5µM (black) and TE buffer (grey) **(A)** without thiourea or **(B)** with thiourea added at 5µM. Non-parametric t test was applied for each time point, p = 0.25 for 2h; 0.88 for 4h; 0.88 for 6h and 0.66 for 24h. ns, non significant p value.

The possible effects of the aptamer bound at the cell surface was studied by two *in vitro* techniques. Initially, the aptamer solution at 10µM, 1µM, 0.1µM, and 0.01µM was inoculated on the bacterial layer but was not able to inhibit the bacterial growth even when thiourea was added to inhibit the action of possible DNases in the medium. Subsequently, time-kill assay showed that the aptamer binding does not seem to interfere with the *A. baumannii* cell growth after 2h (7.29E+00UFC_Normalized_ x 6.00E+00 UFC _Normalized_; p = 0.343), 4h (1.05E+02 x 1.24E+02; p= 0.886), 6h (1.76E+02 x 1.06E+02; p= 0.343), and 24h (5.88E+02 x 1.45E+03; p=0.700) (**Figure 6A**); neither with thiourea after 2h (7.83E+00 x 1.22E+01; p=1.0), 4h (4.85E+01 x 4.05E+01; p= 1.0), 6h (7.56E+01 x 1.16E+02; p= 0.7), and 24h (3.41E+03 x 3.40E+03; p=0.653) (**Figure 6B**).

## Discussion

Here we have modified and standardized the aptamer selection technique, targeting whole-cell (cell-SELEX) with a cost reduction and lower equipment demand, when compared with previously published methodologies (Sefah et al., 2010; Kim et al., 2013; Heiat et al., 2017) which permitted us to identify the first aptamer A01. The new categorized aptamer (A01) displayed significant binding affinity to MDR *A. baumannii* when compared to *K. pneumoniae* during the negative selection performed after the second, fourth, and last rounds of SELEX protocol, as well as during the flow cytometry experiment.

With the constant escalation of antibiotic resistance and high demand for rapid diagnosis techniques, the study of aptamers has been constantly increasing in the field and showing promising results (Boucher et al., 2009). However, aptamer technologies still encounter many challenges including the difficulty of the screening process (Rasoulinejad and Gargari, 2016; Zou et al., 2019). An unusual issue, faced during our experiments pertains to PCR bias, associated with reaction residues and unspecific binders (Rozenblum et al., 2016). Once categorized, the promising aptamers can be modified for better fitness *in vivo*, which include pegylation to improve affinity or adding nuclease-resistant bases, such as locked nucleic acids and 2′-O-methyl nucleotide analogs for nuclease resistance and metabolic stability (Zou et al., 2018; Li et al., 2020).

In the whole-cell SELEX approach, live cells were used for selection and targets were presented in their natural 3D structure. The conservation of this native conformation is essential for the functionality of the selected aptamers, since aptamers present greater affinity to viable cells when compared to inactivated cells (Rasoulinejad and Gargari, 2016). For this reason, aptamers selected with a purified protein may not recognize it in natural conditions (Rasoulinejad and Gargari, 2016).

Combining the previous knowledge with nucleic acid base-pairing and advances in sequencing, structural, and computational technologies, it is possible to develop a low-cost technique with well-defined construction rules to be easily reproduced. During the process we prioritized conserving the basic parameters for a successful SELEX, including pH, temperature, binding settings, aptamer concentration and conformation (Zhou et al., 2017).

Here, we presented an alternative cost-effective methodology, pricing around $300 per aptamer sequence, which may facilitate aptamer research and expand it to low-incoming regions, where the project budget can be a limiting factor in scientific research. The average cost for an aptamer selection and identification is about $4,000 for each individual aptamer sequences (Zou et al., 2019).

Among the limitations of our study, the isolation of high affinity aptamers for gram-negative bacteria was challenging, since the negatively charged outer membrane of these bacteria repels negatively charged nucleic acids (Hamula et al., 2015). Another disadvantage of the aptamer selection is that the success of the selection cannot be predicted, leading to several time-consuming attempts. However, the technology has been increasingly applied in several areas with favorable outcomes.

Additionally, the aptamer A01 demonstrated a binding preference to *A. baumannii* when compared to *K. pneumonia, C albicans*, and *S. aureus* in fluorescence assays, with potential use as a diagnostic tool. Indeed, an electromagnetically driven microfluidic system for *A. baumannii* detection based on aptamers was recently described (Su et al., 2020). Although the time-kill assay did not show an effect on bacterial growth, the potential bactericidal or bacteriostatic cannot be totally discarded. Lee and coworkers already demonstrated that an antimicrobial peptide loaded onto a gold nanoparticle-DNA aptamer conjugate but neither the aptamer nor the antimicrobial peptide alone have an effective therapeutic effect (Lee et al., 2017).

In conclusion, a new DNA aptamer (A01) that demonstrated a binding preference to *A. baumannii* was successfully selected and identified through our in-house modified cell-SELEX protocol, with potential use in diagnosis and/or treatment.

## Data Availability Statement

The original contributions presented in the study are included in the article/supplementary material. Further inquiries can be directed to the corresponding author.

## Author Contributions

MFC, TMB, SFC, and CS conceived and designed the experiments. MFC, TMB, and SFC wrote the manuscript. MFC and AJG analyzed the data. ECS and SFC provided the finacial support for this work execution. MFC, AJG, TMB, BRD and BSA performed the experiments. All authors contributed to the article and approved the submitted version.

## Funding

This work was funded by Fundação de Amparo à pesquisa do estado de São Paulo (FAPESP) (project number 2018/09971-2).

## Conflict of Interest

The authors declare that the research was conducted in the absence of any commercial or financial relationships that could be construed as a potential conflict of interest.

## Publisher’s Note

All claims expressed in this article are solely those of the authors and do not necessarily represent those of their affiliated organizations, or those of the publisher, the editors and the reviewers. Any product that may be evaluated in this article, or claim that may be made by its manufacturer, is not guaranteed or endorsed by the publisher.

## References

[B1] Afzal H. Zahid K. Ali Q. Sarwar K. Shakoor S. Nasir U.. (2016). Role of Biotechnology in Improving Human Health. J. Mol. Biomark. Diagn. 07, 1–7. doi: 10.4172/2155-9929.1000309

[B2] Bellaousov S. Reuter J. S. Seetin M. G. Mathews D. H. (2013). RNAstructure: Web Servers for RNA Secondary Structure Prediction and Analysis. Nucleic Acids Res. 41, W471–W474. doi: 10.1093/nar/gkt290 23620284 PMC3692136

[B3] Boucher H. W. Talbot G. H. Bradley J. S. Edwards J. E. Gilbert D. Rice L. B.. (2009). Bad Bugs, No Drugs : No ESKAPE! Update Infect. Dis. Soc. America 02111, 1–12. doi: 10.1086/595011 19035777

[B4] de Maio Carrillho C. M. D. Gaudereto J. J. Martins R. C. R. de Castro Lima V. A. C. de Oliveira L. M. Urbano M. R.. (2017). Colistin-Resistant Enterobacteriaceae Infections: Clinical and Molecular Characterization and Analysis of *In Vitro* Synergy. Diagn. Microbiol. Infect. Dis. 87, 253–257. doi: 10.1016/j.diagmicrobio.2016.11.007 27939820

[B5] Hamula C. L. A. Peng H. Wang Z. Newbigging A. M. Tyrrell G. J. Li X.-F.. (2015). The Effects of SELEX Conditions on the Resultant Aptamer Pools in the Selection of Aptamers Binding to Bacterial Cells. J. Mol. Evol. 81, 194–209. doi: 10.1007/s00239-015-9711-y 26538121

[B6] Heiat M. Ranjbar R. Latifi A. M. Rasaee M. J. Farnoosh G. (2017). Essential Strategies to Optimize Asymmetric PCR Conditions as a Reliable Method to Generate Large Amount of ssDNA Aptamers. Biotechnol. Appl. Biochem. 64, 541–548. doi: 10.1002/bab.1507 27222205

[B7] Kim L. H. Yu H. W. Kim Y. H. Kim I. S. Jang A. (2013). Potential of Fluorophore Labeled Aptamers for *Pseudomonas Aeruginosa* Detection in Drinking Water. J. Korean Soc. Appl. Biol. Chem. 56, 165–171 doi: 10.1007/s13765-013-3019-7

[B8] Lee B. Park J. Ryu M. Kim S. Joo M. Yeom J.. (2017). Antimicrobial Peptide-Loaded Gold Nanoparticle-DNA Aptamer Conjugates as Highly Effective Antibacterial Therapeutics Against *Vibrio Vulnificus* . Sci. Rep. 7 (1), 13572. doi: 10.1038/s41598-017-14127-z 29051620 PMC5648795

[B9] Leite G. C. Oliveira M. S. Perdigão-Neto L. V. Rocha C. K. D. Guimarães T. Rizek C.. (2016). Antimicrobial Combinations Against Pan-Resistant *Acinetobacter Baumannii* Isolates With Different Resistance Mechanisms. PLoS One 11, e0151270. doi: 10.1371/journal.pone.0151270 26998609 PMC4801211

[B10] Li H. Y. Jia W. N. Li X. Y. Zhang L. Liu C. Wu J. (2020). Advances in Detection of Infectious Agents by Aptamer-Based Technologies. Emerg. Microbes Infect. 9, 1671–1681. doi: 10.1080/22221751.2020.1792352 32623963 PMC7473197

[B11] Pacheco A. B. Guth B. E. Soares K. C. Nishimura L. de Almeida D. F. Ferreira L. C. (1997). Random Amplification of Polymorphic DNA Reveals Serotype-Specific Clonal Clusters Among Enterotoxigenic Escherichia Coli Strains Isolated From Humans. J. Clin. Microbiol. 35, 1521–1525. doi: 10.1128/jcm.35.6.1521-1525.1997 9163473 PMC229778

[B12] Petersen P. J. Labthavikul P. Jones C. H. Bradford P. A. (2006). *In Vitro* Antibacterial Activities of Tigecycline in Combination With Other Antimicrobial Agents Determined by Chequerboard and Time-Kill Kinetic Analysis. J. Antimicrob. Chemother. 57, 573–576. doi: 10.1093/jac/dki477 16431863

[B13] Rasoulinejad S. Gargari S. L. M. (2016). Aptamer-Nanobody Based ELASA for Specific Detection of *Acinetobacter Baumannii* Isolates. J. Biotechnol. 231, 46–54. doi: 10.1016/j.jbiotec.2016.05.024 27234880

[B14] Rozenblum G. T. Lopez V. G. Vitullo A. D. Radrizzani M. (2016). Aptamers: Current Challenges and Future Prospects. Expert Opin. Drug Discov. 11, 127–135. doi: 10.1517/17460441.2016.1126244 26630462

[B15] Sefah K. Shangguan D. Xiong X. O’Donoghue M. B. Tan W. (2010). Development of DNA Aptamers Using Cell-SELEX. Nat. Protoc. 5, 1169–1185. doi: 10.1038/nprot.2010.66 20539292

[B16] Su C. H. Tsai M. H. Lin C. Y. Ma Y. D. Wang C. H. Chung Y. D.. (2020). Dual Aptamer Assay for Detection of *Acinetobacter Baumannii* on an Electromagnetically-Driven Microfluidic Platform. Biosens Bioelectron 59, 112148. doi: 10.1016/j.bios.2020.112148 32291246

[B17] Tuerk C. Gold L. (1990). Systematic Evolution of Ligands by Exponential Enrichment: RNA Ligands to Bacteriophage T4 DNA Polymerase. Science 80-) 249, 505–510. doi: 10.1126/science.2200121 2200121

[B18] Ulrich H. Martins A. H. B. Pesquero J. B. (2005). RNA and DNA Aptamers in Cytomics Analysis. Cytometry 59A, 220–231. doi: 10.1002/0471142956.cy0728s33 15170601

[B19] Zhou L. Li P. Ni S. Yu Y. Yang M. Wei S.. (2017). Rapid and Sensitive Detection of Redspotted Grouper Nervous Necrosis Virus (RGNNV) Infection by Aptamer–Coat Protein–Aptamer Sandwich Enzyme-Linked Apta-Sorbent Assay (ELASA). J. Fish Dis. 40, 1831–1838. doi: 10.1111/jfd.12656 28745819

[B20] Zou Y. Duan N. Wu S. Shen M. Wang Z. (2018). Selection, Identification, and Binding Mechanism Studies of an ssDNA Aptamer Targeted to Different Stages of E. Coli O157:H7. J. Agric. Food Chem. 66, 5677–5682. doi: 10.1021/acs.jafc.8b01006 29756774

[B21] Zou X. Wu J. Gu J. Shen L. Mao L. (2019). Application of Aptamers in Virus Detection and Antiviral Therapy. Front. Microbiol. 10, 1462. doi: 10.3389/fmicb.2019.01462 31333603 PMC6618307

